# Poly-L-lactic acid (PLLA) in arm contouring: a prospective and blinded trial of Rennova® Elleva and Sculptra®

**DOI:** 10.3389/fsurg.2026.1782368

**Published:** 2026-05-08

**Authors:** Bruna Souza Felix Bravo, Ana Luisa Leopoldino de Souza, Emmanuela Beatriz Vantini Barreiro, Airá Novello Vilar, Bárbara Fouraux Gouvea, Leonardo Gonçalves Bravo, Marina Ramos Baeta Neves

**Affiliations:** 1 Bravo Private Clinic, Rio de Janeiro, Rio de Janeiro, Brazil; 2 Ferreira Vilar Private Clinic, Concórdia, Santa Catarina, Brazil

**Keywords:** aesthetic outcomes, arm rejuvenation, biostimulatory fillers, dermal remodeling, ultrasound

## Abstract

**Background:**

Poly-L-lactic acid (PLLA) is a biostimulatory filler used to improve dermal laxity and contour, yet comparative data between formulations for upper-arm rejuvenation remain scarce.

**Methods:**

In this prospective, randomized, blinded split-arm trial, 20 adults received bilateral upper-arm injections with two PLLA formulations (Rennova® Elleva vs. Sculptra®). Outcomes included Subject and Physician Global Aesthetic Improvement Scales (S-GAIS, P-GAIS), ultrasonography (USG), skin elasticity, and transepidermal water loss (TEWL) through Day 120. Histological analyses in ten cases assessed skin thickness, inflammation, and crystalline residues.

**Results:**

Both products yielded marked enhancements in skin texture and arm contour with excellent tolerability. S-GAIS scores improved to 2.9 ± 0.67 at Day 120, with no differences between formulations (*p* > 0.99). P-GAIS scores similarly rose (Rennova® Elleva: 3.2 ± 0.98; Sculptra®: 3.3 ± 0.90; *p* = 0.86). Ultrasound findings confirmed dermal remodeling in both arms. By Day 120, USG value increased to 957 ± 209 with Rennova® Elleva (*p* = 0.049) and 938 ± 174 with Sculptra® (*p* = 0.92), with no between-group difference (*p* = 0.99). Skin elasticity increased significantly in both arms by Day 90 (Rennova® Elleva: 172 ± 35 to 191 ± 29, *p* = 0.035; Sculptra®: 165 ± 19 to 177 ± 24, *p* = 0.041), while TEWL remained stable. Skin thickness rose significantly (Rennova® Elleva: 2.06 ± 0.55 to 2.66 ± 0.53 mm, *p* = 0.039; Sculptra®: 1.80 ± 0.41 to 2.70 ± 0.69 mm, *p* = 0.0039), without between-group differences (*p* = 0.54). Histology showed sparse perivascular lymphocytes and occasional macrophages, with no granulomas or crystalline material.

**Conclusions:**

This study suggests that both Rennova® Elleva and Sculptra® are effective, well-tolerated PLLA formulations that may be capable of producing meaningful aesthetic and biophysical improvements in upper-arm contour and skin quality within the first 120 days.

## 1 Introduction

Poly-L-lactic acid (PLLA) is a synthetic, biodegradable, injectable polymer that has been used in aesthetic medicine for nearly three decades (1, 2). PLLA improves contour and skin quality by stimulating neocollagenesis and elastin synthesis through activation of endogenous regenerative pathways (1, 3, 4). Its biostimulatory mechanism leads to gradual, natural-appearing improvements, making it particularly appealing for patients seeking subtle yet meaningful aesthetic enhancement (1, 2). According to the 2024 International Society of Aesthetic Plastic Surgery (ISAPS) report, 642,566 procedures involving PLLA were performed worldwide, representing a substantial increase from previous years and underscoring the rapidly growing demand for biostimulatory injectables (5).

Although PLLA was initially used predominantly for facial rejuvenation, its applications have expanded significantly (6–8). Today, tissue stimulators play a central role in non-surgical body contouring, offering an effective means of improving skin laxity, restoring youthful appearance, and addressing post-surgical contour deformities (7, 8). The upper arm, in particular, has emerged as an increasingly common treatment site due to its susceptibility to age-related thinning of the dermis, decreased elasticity, and contour irregularities (9). For many patients, these changes tend to be resistant to diet, exercise, and conventional skin-tightening interventions, positioning PLLA as a valuable minimally invasive alternative to surgical procedures such as brachioplasty (6, 7).

Despite the rising clinical use of PLLA for body applications, the current literature continues to highlight a need for high-quality evidence assessing treatment outcomes, longevity, and product-specific performance (8, 10). Large expert panels have emphasized the importance of studies focused on optimizing injection protocols, dilution volumes, and treatment regimens for PLLA in the upper arms (8, 10). Current recommendations suggest an initial dilution volume of approximately 17 mL per vial and the use of a 25–26-gauge needle or a 22-gauge cannula, typically administered over 2–4 sessions with a minimum interval of at least 30 days (10). While these guidelines provide a valuable framework, they are largely based on consensus rather than direct comparative evidence (8).

Multiple PLLA formulations exist on the global market, and differences in particle size, lyophilization processes, excipients, and reconstitution characteristics may influence clinical performance (1, 7). Comparative studies are therefore critical for informing product selection and guiding treatment (7, 8). Although a previous study has compared two widely used PLLA products—Elleva® and Sculptra®—in the upper arm, its evaluations were limited to immediate post-injection distribution (11). Long-term follow-up, which is essential for understanding biostimulatory efficacy, tissue integration, and adverse-event development, was not included (11).

To address these gaps, we conducted a prospective, blinded interventional study comparing the clinical performance of Rennova® Elleva and Sculptra® specifically for upper-arm contouring. Twenty patients were enrolled, and each participant received both products, one assigned to each arm, thereby eliminating inter-individual variability and enabling direct, within-patient comparison. Long-term follow-up allowed for comprehensive assessment of aesthetic improvement, tissue quality, durability of effect, and safety profiles. In addition, ultrasound imaging was incorporated to deepen understanding of product distribution and the underlying mechanisms of tissue response over time (11). The objective of this study was to evaluate and compare the clinical and aesthetic outcomes of two commercially available PLLA formulations used for arm contouring. By combining a blinded, split-arm design with long-term evaluation and ultrasound assessment, this investigation provides rigorous evidence that may guide clinicians in treatment planning, product selection, and optimization of PLLA protocols for upper-arm rejuvenation and contour enhancement.

## 2 Materials & methods

### 2.1 Study setup

This prospective, single-center, blinded interventional trial was designed to compare the clinical performance of two PLLA biostimulatory injectables: Rennova® Elleva and Sculptra®. Twenty healthy adult participants underwent bilateral treatment, with one arm randomized to receive Rennova® Elleva and the contralateral arm receiving Sculptra®. This intra-individual design minimized interpersonal variability and ensured a controlled and direct comparison under blinded conditions. Both participants and clinical evaluators remained blinded to product allocation throughout the study period. The study was conducted between April 2025 and October 2025 at the senior author's private practice. Written informed consent was obtained from all participants. The trial adhered to the ethical standards outlined in the 1996 Declaration of Helsinki, conformed to applicable regulatory requirements, and followed Good Clinical Practice guidelines. Institutional ethics approval was obtained from the Research Ethics Committee of Hospital Universitário Clementino Fraga Filho/Universidade Federal do Rio de Janeiro (CEP HUCFF/FM/UFRJ), under approval number 7.259.645. The primary outcome measure was global aesthetic improvement as assessed by the Subject Global Aesthetic Improvement Scale (S-GAIS) and the Physician Global Aesthetic Improvement Scale (P-GAIS). Secondary endpoints included ultrasonographic parameters (USG Value), skin elasticity, transepidermal water loss (TEWL), skin thickness, and histological findings. No formal sample size or power calculation was performed. This is acknowledged as a limitation, and the potential for type II error, particularly in between-group comparisons, should be considered when interpreting the results.

### 2.2 Inclusion and exclusion criteria

Eligible individuals were healthy adults aged 18 years or older. Participants were excluded if pregnant or breastfeeding, given the potential risks to mother and child. Individuals with a history of autoimmune disease were excluded to avoid confounding immune-mediated responses, and the use of systemic immunosuppressive or anti-inflammatory medications constituted an exclusion criterion to prevent interference with treatment outcomes. Participants using topical or systemic agents known to alter skin physiology, such as retinoids, corticosteroids, or other collagen-modulating compounds, were also excluded to maintain sample homogeneity and ensure unbiased evaluation of the biostimulatory effects. Patients who had undergone any invasive or major treatment, including procedures involving the arms, within the 12 months preceding study enrollment were not eligible.

### 2.3 Product specifications and procedure

Rennova® Elleva is a sterile, pyrogen-free, bioresorbable poly-L-lactic acid (PLLA) injectable indicated for facial and body biostimulation. Each vial contains 150 mg PLLA, formulated as a lyophilized powder requiring reconstitution before injection. The formulation includes poly-L-lactic acid particles, carboxymethylcellulose (CMC) as a suspending agent, and mannitol as a stabilizing excipient. Sculptra® (Galderma) is an FDA-approved sterile lyophilized formulation of 150 mg PLLA, combined with sodium carboxymethylcellulose and non-pyrogenic mannitol to facilitate suspension and maintain particle stability.

Both Rennova® Elleva and Sculptra® were reconstituted according to manufacturer recommendations and standardized across all patients. A final suspension concentration of 8.3 mg/mL was prepared by mixing sterile water for injection with 2 mL of 2% lidocaine, resulting in a total volume of 18 mL per vial. The reconstituted materials were administered subcutaneously using a 22-G × 50 mm cannula employing fanning injection techniques to ensure comparability between arms. All procedures were performed by two experienced injectors (BSFB and MRBN). Gentle post-injection massage was conducted to promote uniform particle dispersion and mitigate the risk of nodule formation.

### 2.4 Global aesthetic improvement scale (GAIS)

Aesthetic outcomes were assessed using both the Subject Global Aesthetic Improvement Scale (S-GAIS) and the Physician Global Aesthetic Improvement Scale (P-GAIS). The S-GAIS was completed by participants at days 30, 60, 90, and 120 post-treatment to evaluate perceived global aesthetic change relative to baseline. This five-point ordinal scale ranges from “exceptionally improved” (1) to “worse” (5), capturing the participant's overall subjective impression rather than isolated aesthetic features. Importantly, lower scores reflect greater improvement. On day 120, two independent physicians, blinded to product allocation, assessed both arms using the P-GAIS. The identical five-point scale was applied, allowing for a structured comparison between patient-reported outcomes and physician-determined aesthetic improvement.

### 2.5 Ultrasonographic and biophysical assessments

#### 2.5.1 Ultrasonography

Ultrasonographic (USG) assessments were performed using the DermaLab® Combo-4 system (Cortex Technology, Aalborg, Denmark) at baseline (day 0) and on days 30, 60, 90, and 120. Ultrasound echogenicity (USG Value) represents the degree of acoustic backscatter within the dermis and serves as an indirect surrogate marker for collagen density and general dermal matrix organization. Participants were positioned at a 45° angle to minimize gravitational distortion, and a thick layer of Aquasonic Clear ultrasound gel was applied to ensure optimal acoustic coupling without mechanical compression. All measurements were obtained using standardized device settings to maintain reproducibility across visits and treatment arms.

#### 2.5.2 Skin elasticity

Skin elasticity was assessed bilaterally at four time points (Days 0, 30, 60, and 90) using the DermaLab® Skin Elasticity Probe. This device measures the vertical elevation of the skin induced by a controlled suction force and records the subsequent retraction time once the suction is released. The resulting parameter reflects the skin's ability to deform and return to its original position, providing a quantitative measure of elastic recoil. As elasticity decreases with intrinsic aging and photoaging, longer retraction times indicate impaired biomechanical skin properties.

The DermaLab® system performs the measurement by applying a standardized negative pressure through a small suction chamber placed perpendicular to the skin surface. The device's software instantaneously calculates elasticity parameters based on deformation and recovery kinetics. All measurements were performed according to manufacturer guidelines and in a temperature-controlled environment after a minimum acclimatization period of 15 min.

#### 2.5.3 Transepidermal water loss (TEWL)

Transepidermal water loss (TEWL) was evaluated to assess changes in epidermal barrier function over time. Using the DermaLab® TEWL probe, measurements were taken bilaterally at days 0, 30, 60, 90, and 120. Stable or decreased TEWL values typically reflect preserved or improved barrier integrity, whereas an increase may indicate barrier disruption or inflammatory alterations. TEWL served as a quantitative marker of treatment-induced changes in epidermal physiology.

### 2.6 Histological and histopathological analysis

In ten randomly selected participants, 4-mm punch biopsies were obtained from the proximal–medial portion of both the right and left upper arms. Sampling was performed at two time points: D0, immediately before product administration, and D120, 120 days after treatment. All tissue specimens were processed and stained with hematoxylin and eosin (H&E). An independent board-certified pathologist examined multiple histological sections from each sample to assess for the presence of crystalline material, foreign-body residues, or granulomatous reactions. Because punch biopsy inherently captures only a limited fraction of the injected region, the resulting histological analyses reflect localized tissue responses rather than a full representation of the entire treatment area.

In addition to the qualitative assessment, dermal thickness was systematically measured in all biopsy samples using a standardized, protocol-driven histomorphometric approach. Measurements were performed under consistent magnification and orientation criteria to ensure reproducibility and minimize operator-dependent variability.

### 2.7 Statistical analysis

Statistical analyses were performed using IBM SPSS Statistics version 27. Within-group comparisons between baseline (Day 0) and each follow-up time point were conducted using the Wilcoxon signed-rank test. Between-group comparisons at follow-up were assessed using the Mann–Whitney U test. A two-sided *p*-value ≤ 0.05 was considered statistically significant. Data are presented as medians with interquartile ranges (IQR), unless otherwise specified.

## 3 Results

### 3.1 Patients characteristics

Twenty female participants were enrolled. The mean age was 51 years (range, 32–65), with a mean body weight of 60.7 kg (range, 47.6–78) and a mean height of 1.63 m (range, 1.50–1.78). Two participants reported allergies (adhesive tape, sulfonamides). Most subjects had undergone previous surgical procedures, most commonly cesarean delivery and aesthetic surgeries such as abdominoplasty, mammoplasty, and liposuction. No participant had a prior history of arm surgery.

### 3.2 Clinical outcomes

**Figure 1:**
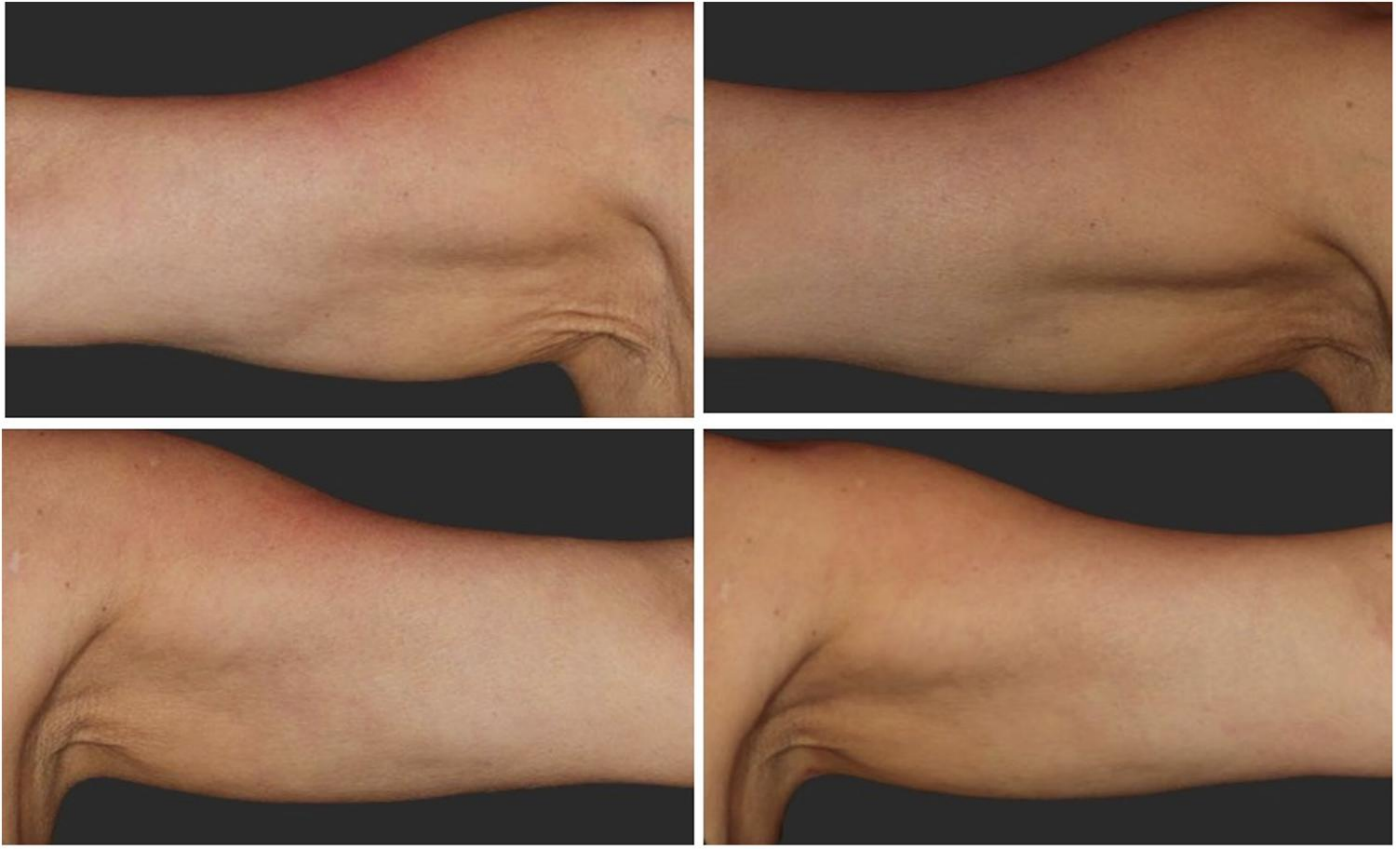
Standardized before-and-after clinical photographs demonstrating upper-arm outcomes following PLLA treatment. Top left: right arm before treatment. Top right: right arm after treatment. Bottom left: left arm before treatment. Bottom right: left arm after treatment. Images show noticeable improvements in skin texture, firmness, and overall contour.

Both treatments demonstrated a favorable safety profile with marked improvements in skin texture and arm contour (Figure 1). No procedure-related adverse events of clinical relevance were observed. Transient ecchymosis and mild post-injection discomfort were the only reported effects, and all resolved spontaneously without intervention.

### 3.3 GAIS data

Mean S-GAIS scores were 3.45 ± 0.69 at Day 30, 3.33 ± 0.73 at Day 60, and 3.25 ± 0.58 at Day 90. By Day 120, both products yielded a mean S-GAIS of 2.9 ± 0.67, corresponding to an “improved” appearance. No significant difference in S-GAIS was observed between Rennova® Elleva and Sculptra® (*p* > 0.99) (Table 1).

**Table 1:** Global aesthetic improvement scores at Day 120 for Elleva® and Sculptra®.

Outcome	Product	Day 120 Mean ± SD	Day 120 Median (IQR)	Elleva vs. Sculptra *p*-Value (Mann–Whitney)
SGAIS	Elleva	2.9 ± 0.67	3 (IQR: 2–3)	>0.99
	Sculptra	2.9 ± 0.67	3 (IQR: 2–3)	
PGAIS	Elleva	3.2 ± 0.98	3 (IQR: 2.5–4)	0.86
	Sculptra	3.3 ± 0.9	3 (IQR: 3–4)	

Comparison of patient- and physician-rated aesthetic improvement 120 days after treatment with Elleva® and Sculptra®. Outcomes include the Subject Global Aesthetic Improvement Scale (SGAIS) and Physician Global Aesthetic Improvement Scale (PGAIS). Data are presented as mean ± standard deviation (SD) and median with interquartile range (IQR). SGAIS, subject global aesthetic improvement scale; PGAIS, physician global aesthetic improvement scale; SD, standard deviation; IQR, interquartile range.

At Day 120, physician evaluators rated both treatment sites comparably. Median P-GAIS scores were 3 for both products, with IQRs of 2.5–4 for Rennova® Elleva and 3–4 for Sculptra®. Mean values were 3.2 ± 0.98 and 3.3 ± 0.90, respectively. No significant between-group differences were noted (*p* = 0.86) (Table 1).

### 3.4 Ultrasound-derived and biophysical skin assessment

#### 3.4.1 Baseline comparisons

At Day 0, the two study groups demonstrated comparable biophysical profiles. Mean USG Values were 797 ± 374 for Rennova® Elleva and 860 ± 371 for Sculptra®, with corresponding median values of 856 (IQR 610–961) and 948 (IQR 777–1,053), respectively. Skin elasticity values at baseline were similarly aligned, with Rennova® Elleva presenting a mean of 172 ± 35 and a median of 162 (IQR 145–195), while Sculptra® showed 165 ± 19 and a median of 167 (IQR 149–169). Baseline TEWL values were also nearly identical, measuring 3.9 ± 1.9 with a median of 4.02 (IQR 2.6–4.36) for Rennova® Elleva, and 4.0 ± 2.3 with a median of 3.43 (IQR 2.56–4.66) for Sculptra®. No statistically significant differences were observed between the two groups at baseline for any of the parameters (Table 2).

**Table 2:** Comparison of biophysical skin parameters between Elleva® and Sculptra® at baseline and follow-up.

Outcome	Product	Day 0 Mean ± SD	Day 0 Median (IQR)	Day 120 Mean ± SD	Day 120 Median (IQR)	Day 0 vs. 120 *p*-Value (Wilcoxon)	Elleva vs. Sculptra *p*-Value (Mann–Whitney)
USG Value	Elleva	797 ± 374	856 (IQR: 610–961)	957 ± 209	961 (IQR: 829.5–1,080)	0.049	0.99
	Sculptra®	860 ± 371	948 (IQR: 777–1,053)	938 ± 174	974 (IQR: 796.5–1,027)	0.92	
Skin Elasticity^a^	Elleva	172 ± 35	162 (IQR: 145–195)	191 ± 29	186 (IQR: 174–203)	0.035	0.29
	Sculptra®	165 ± 19	167 (IQR: 149–169)	177 ± 24	177 (IQR: 170–187)	0.041	
TEWL	Elleva	3.9 ± 1.9	4.02 (IQR: 2.6–4.36)	3.7 ± 1.3	4.225 (IQR: 3.14–4.56)	0.58	0.94
	Sculptra®	4 ± 2.3	3.43 (IQR: 2.56–4.66)	3.9 ± 1.3	4.17 (IQR: 3.23–4.595)	0.83	
Skin Thickness	Elleva	2.06 ± 0.55	2.1 (IQR: 1.56–2.35)	2.66 ± 0.53	2.65 (IQR: 2.56–2.65)	0.039	0.54
	Sculptra®	1.8 ± 0.41	1.8 (IQR: 1.58–1.98)	2.7 ± 0.69	2.7 (IQR: 2.12–2.97)	0.0039	

Comparison of ultrasound-derived echogenicity (USG Value), skin elasticity, transepidermal water loss (TEWL), and skin thickness between Rennova® Elleva and Sculptra® at baseline (Day 0) and follow-up. USG Value, USG Intensity, TEWL, and skin thickness were assessed at Day 120, whereas skin elasticity was evaluated at Day 90 due to missing follow-up data at Day 120. Data are presented as mean ± standard deviation (SD) and median with interquartile range (IQR). USG, ultrasound; USG Value: ultrasound echogenicity; TEWL: transepidermal water loss; IQR: interquartile range; SD: standard deviation.

a Skin elasticity was analyzed day 0 to day 90 due to missing follow-up entries at day 120.

#### 3.4.2 Longitudinal changes within groups

Both products produced measurable changes from baseline to follow-up, although the magnitude and statistical significance differed by outcome measure. For Rennova® Elleva, USG Value increased from 797 ± 374 at baseline to 957 ± 209 at Day 120, with median values rising from 856 (IQR 610–961) to 961 (IQR 829.5–1,080), a statistically significant change (*p* = 0.049). Sculptra® demonstrated a smaller and statistically non-significant shift, increasing from 860 ± 371 to 938 ± 174, with medians increasing from 948 (IQR 777–1,053) to 974 (IQR 796.5–1,027) (*p* = 0.92) (Table 2). Further details can be found in Supplementary Table 1.

Skin elasticity improved significantly in both groups between Day 0 and Day 90: Rennova® Elleva increased from a baseline mean of 172 ± 35 to 191 ± 29 at Day 90, with medians shifting from 162 (IQR 145–195) to 186 (IQR 174–203) (*p* = 0.035). Sculptra® similarly rose from 165 ± 19 to 177 ± 24, and its medians increased from 167 (IQR 149–169) to 177 (IQR 170–187) (*p* = 0.041) (Table 2). TEWL remained stable in both groups. Rennova® Elleva showed a non-significant change from 3.9 ± 1.9 to 3.7 ± 1.3, with median values of 4.02 (IQR 2.6–4.36) and 4.225 (IQR 3.14–4.56) (*p* = 0.58). For Sculptra®, TEWL changed minimally from 4.0 ± 2.3 to 3.9 ± 1.3, with medians of 3.43 (IQR 2.56–4.66) and 4.17 (IQR 3.23–4.595) (*p* = 0.83) (Table 2).

#### 3.4.3 Between-group comparisons

Despite significant within-group improvements in some parameters, between-groups comparisons demonstrated no statistically significant differences at follow-up. USG Values at Day 120 did not differ between Rennova® Elleva and Sculptra® (*p* = 0.99). Similarly, no significant differences were detected in skin elasticity at Day 90 (*p* = 0.29). TEWL values at Day 120 also remained comparable between the two products (*p* = 0.94). Thus, although Rennova® Elleva demonstrated a statistically significant increase in echogenicity at follow-up and slightly more pronounced gains in elasticity, these differences did not translate into measurable superiority over Sculptra® when evaluated using nonparametric between-group testing (Table 2).

### 3.5 Skin thickness and histopathological analysis

At baseline, cases with subsequent Rennova® Elleva treatment demonstrated a mean dermal thickness of 2.06 ± 0.55 mm with a median of 2.1 (IQR 1.56–2.35), while Sculptra® presented 1.80 ± 0.41 mm and a median of 1.8 (IQR 1.58–1.98). There was no statistically significant difference between these initial values.

**Figure 2:**
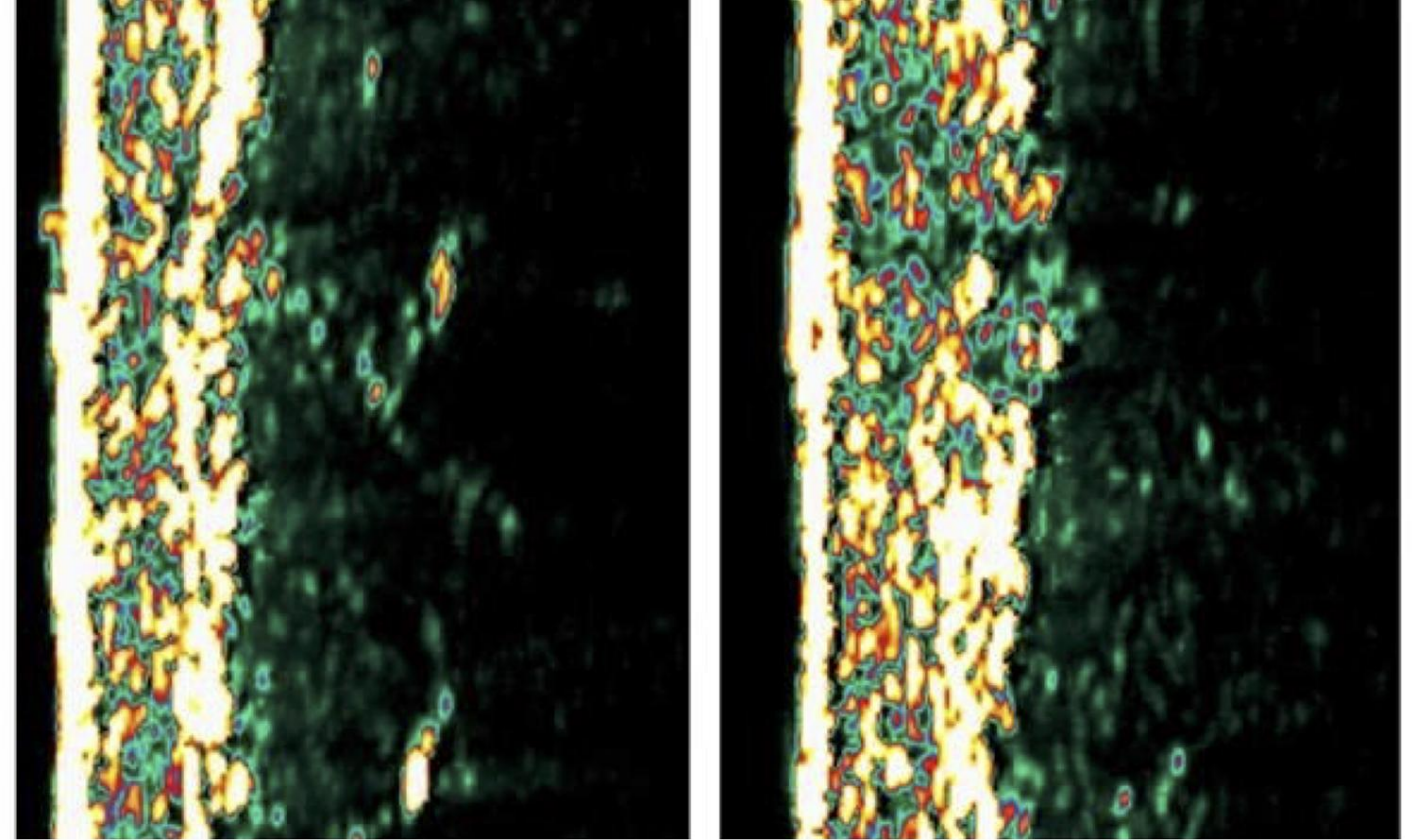
Ultrasound imaging of the upper arm. Left: pre-treatment baseline. Right: post-treatment assessment demonstrating changes in dermal structure following PLLA administration.

Over the 120-day interval, both products produced statistically significant increases in skin thickness. Rennova® Elleva increased to 2.66 ± 0.53 mm, with a median of 2.65 (IQR 2.56–2.65), representing a significant change from baseline (*p* = 0.039). Sculptra® increased to 2.70 ± 0.69 mm, with a median of 2.7 (IQR 2.12–2.97), also demonstrating a statistically significant increase (*p* = 0.0039). The magnitude of dermal expansion did not differ between groups (*p* = 0.54) (Table 2, Figure 2).

**Figure 3:**
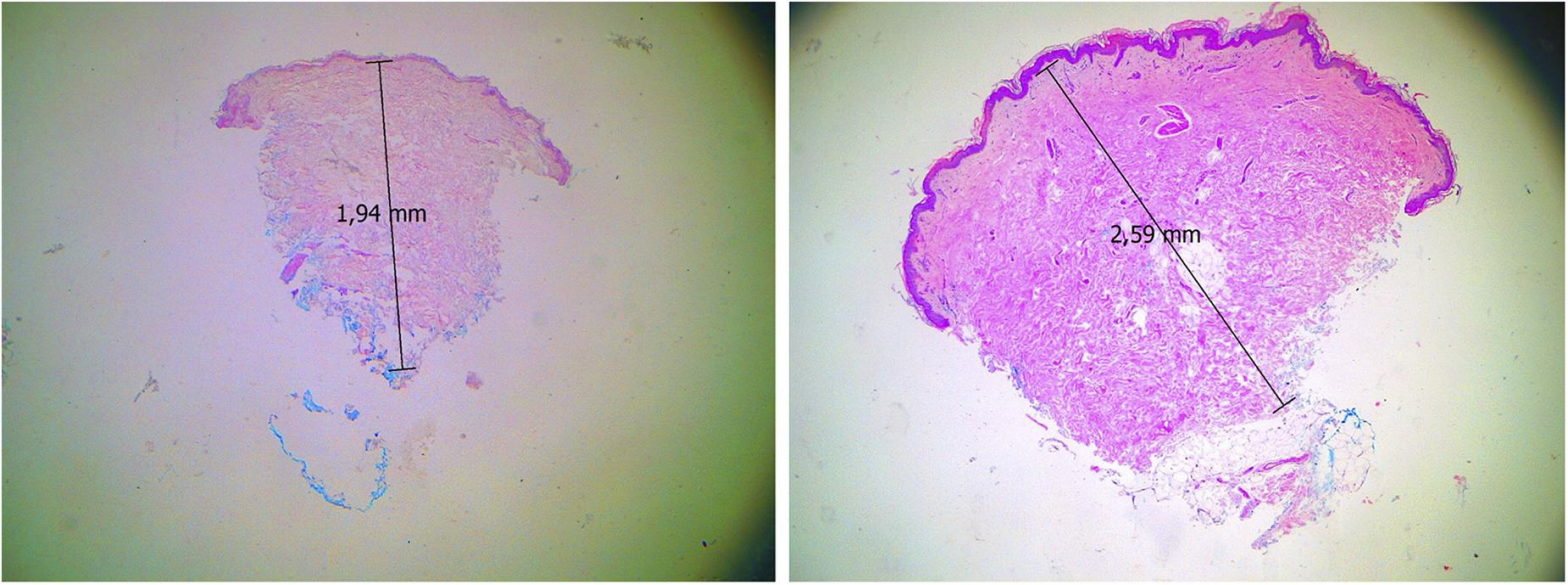
Representative before-and-after H&E-stained sections of biopsy specimens. The analyzed tissue fragments measure approximately 1.94 mm (left; before PLLA treatment) and 2.59 mm (right; after PLLA treatment) at their greatest dimensions. No significant inflammatory infiltrate, granuloma formation, or foreign material compatible with injected biomaterials was detected.

Histopathological evaluation of H&E-stained sections revealed no significant inflammatory infiltrate in either the pre- or post-treatment specimens. Only sparse perivascular inflammatory cells, predominantly lymphocytes with occasional macrophages, were present, and no granuloma formation was observed (Figure 3). The minimal inflammatory response is likely related to the interval between product administration and tissue sampling.

No crystalline deposits or foreign material compatible with either Sculptra® or Rennova® Elleva were identified in any of the examined sections. Despite multiple histological levels being assessed, it remains possible that the analyzed fragments did not encompass the entire injected area. Nevertheless, no residual biomaterial was detected in any of the samples.

## 4 Discussion

The present prospective, blinded, split-arm study provides a comparative and comprehensive evaluation of two commercially available PLLA formulations—Rennova® Elleva and Sculptra®—for upper-arm biostimulation and contour enhancement. By integrating patient-reported outcomes, blinded physician assessments, quantitative ultrasound metrics, biophysical skin measurements, and histological examination, this investigation offers a multidimensional characterization of product performance over a 120-day period. Overall, the findings demonstrate that both formulations yield comparable clinical and biophysical improvements, with no meaningful differences in safety, tolerability, or aesthetic benefit within the study timeframe. These observations should be understood as specific to the 120-day follow-up period, with extrapolation to longer timeframes requiring further investigation with extended observation windows.

Consistent improvement in both S-GAIS and P-GAIS scores confirms that PLLA-based biostimulation in the upper arm produces perceptible aesthetic enhancement across subjective and objective evaluators (Table 1). The convergence of patient and physician assessments underscores the clinical relevance of the observed changes and aligns with the growing body of evidence supporting PLLA as a reliable modality for addressing dermal laxity and contour irregularities in body regions beyond the face (12–15).

Ultrasonographic measurements, widely regarded as robust indicators of dermal remodeling, further substantiate the GAIS data (16, 17). Both products induced significant increases in skin thickness, a hallmark of PLLA-driven neocollagenesis. This is consistent with established histologic data showing progressive fibroblast recruitment, collagen deposition, and extracellular matrix reorganization following PLLA injection (4, 9). Although Rennova® Elleva demonstrated a statistically significant increase in USG value over time whereas Sculptra® did not, the lack of a significant between-group difference suggests that this discrepancy reflects intra-group variability rather than a true divergence in biological activity. Furthermore, USG intensity, an indicator often associated with the evolving echogenicity of collagenous structures, remained stable in both treatment arms across the evaluation period. This stability indicates the early phases of PLLA-induced remodeling, during which collagen deposition is initiated but not yet fully reorganized into higher-order, acoustically distinct structures (18). As collagen bundles progressively align and mature, they may yield detectable shifts in acoustic reflectivity at later time points.

Biophysical assessments provide additional functional insight into the quality and physiological state of the regenerating dermis (Table 2). The significant increases in skin elasticity observed for both formulations indicate improved biomechanical resilience of the treated tissue—a functional correlate of progressive extracellular matrix regeneration. Enhanced elasticity is consistent with early collagen deposition, increased dermal density, and improved organization of elastic fiber networks, all of which contribute to greater tensile recovery and recoil capacity of the skin (19). Importantly, the parallel improvement in elasticity across both treatment arms supports the notion that the two PLLA formulations elicit comparable biostimulatory effects on dermal architecture.

Equally noteworthy is the stability of TEWL values throughout the study period. The preservation of epidermal barrier integrity underscores that the biostimulatory process induced by PLLA occurs without compromising cutaneous homeostasis. This finding is clinically relevant, as it supports the notion that dermal remodeling can be achieved without perturbing the barrier functions essential for protecting against environmental stressors, maintaining hydration, and preventing inflammation. Taken together, these quantitative biophysical findings illustrate not only the structural enhancement of the treated dermis but also its functional strengthening, characterized by increased elastic performance and preserved barrier function.

Histological analyses revealed no granulomatous reactions, crystalline deposits, or residual foreign material in any evaluated sample. While the absence of identifiable PLLA particles is not unexpected given the timing of biopsy acquisition, after substantial hydrolysis and cellular clearance, these findings provide important reassurance regarding tissue compatibility. The minimal inflammatory infiltrate is consistent with contemporary understanding of PLLA's mechanism of action, in which a controlled, subclinical inflammatory response initiates collagen synthesis without eliciting overt or persistent inflammatory pathology (20, 21). Notably, the histologic similarity between products further supports the clinical and biophysical equivalence observed, at least in the early 120-day period. The histological component of this study, however, carries important interpretive limitations that bear directly on the conclusions that can be drawn from it. Punch biopsies are a spatially constrained sampling tool: a 4-mm core captures a cylindrical tissue volume representing only a small fraction of the total subcutaneous area into which PLLA was delivered. Because PLLA particles are intentionally dispersed across a broad plane rather than deposited at a single focal point, the probability that any given biopsy core coincides with a site of meaningful product concentration is inherently low. Therefore, a positive finding would carry strong confirmatory value, whereas a negative finding cannot be taken as evidence of true tissue absence of product. Therefore, the observed absence of crystalline structures or foreign material should be interpreted as a sampling outcome, not as a biological conclusion about the fate of the injected material within the treated tissue. The findings are therefore most appropriately framed as “no adverse histological signal was detected within the sampled tissue” rather than as evidence of total product clearance or confirmed biocompatibility across the treatment area as a whole. Future studies in this area would benefit from larger biopsy panels, higher sampling density, or the use of techniques such as polarised light microscopy or spectroscopic analysis, which offer greater sensitivity for detecting residual biomaterial below the threshold of conventional H&E staining.

Taken together, the results of this study have several implications for clinical practice. First, they affirm that both Rennova® Elleva and Sculptra® are effective and well-tolerated options for upper-arm rejuvenation and contouring, with no evidence favoring one product over the other in early outcomes. Second, the equivalence in early performance despite differences in PLLA mass and formulation characteristics suggests that clinical response may be more closely tied to injection technique, dilution strategies, and patient-specific tissue biology than to intrinsic product differences. This highlights the centrality of standardized protocols and skilled technique in achieving optimal results. Finally, the intra-individual, blinded design strengthens the internal validity of these conclusions and provides a high level of evidence that will be valuable in informing product selection and treatment planning.

## 5 Limitations

This study has several limitations that should be acknowledged. First, the sample size was relatively small and limited to a single center, which may restrict the generalizability of the findings. Additionally, no formal sample size or power calculation was performed prior to study initiation. The sample of 20 participants was determined pragmatically, and the study may therefore be underpowered to detect clinically meaningful differences between treatments, particularly for between-group comparisons. The risk of type II error should accordingly be considered when interpreting the absence of statistically significant between-group differences. Second, no correction for multiple comparisons was applied across secondary outcome measures, which raises the potential for Type I error. Third, the 120-day observation window captures only the early to intermediate phase of PLLA-induced neocollagenesis, and conclusions regarding long-term equivalence, durability, or potential product-specific differences emerging beyond this window cannot be established from the current dataset. Future studies with longer-term follow-up data are planned. Fourth, although the split-arm design minimized inter-individual variability, it cannot fully eliminate systemic or behavioral influences that may affect bilateral tissue responses. Fifth, histological evaluation was based on small, localized biopsy samples, which may not fully represent the broader tissue changes occurring across the entire treated region. Sixth, ultrasound and biophysical measurements, while objective, may be influenced by operator technique and device-specific limitations, and future studies incorporating multimodal imaging could provide a more comprehensive characterization of tissue remodeling. Finally, in several specimens, partial or complete absence of subcutaneous adipose tissue was noted, likely due to fragmentation or detachment during punch rotation, which introduced technical limitations to the precision of dermal thickness measurements. These sampling artifacts may bias quantitative values and therefore must be considered when interpreting pre- to post-treatment differences. Additionally, the absence of detectable product in the subcutaneous layer should not be interpreted as a true absence but rather as a consequence of insufficient tissue sampling. Future research might benefit from longer follow-up intervals, larger sample sizes, and inclusion of advanced imaging modalities, such as elastography, to further delineate the kinetics of tissue remodeling.

## 6 Conclusions

This prospective, blinded, split-arm study provides preliminary evidence that both Rennova® Elleva and Sculptra® are associated with measurable improvements in upper-arm skin quality and contour over a 120-day observation period. Across patient-reported outcomes, physician assessments, ultrasound-derived parameters, and biophysical measurements, both formulations demonstrated consistent trends toward enhanced dermal properties, including increased skin thickness and elasticity, while maintaining epidermal barrier integrity. Importantly, no statistically significant differences between the two products were identified. However, the present study was not designed or powered to establish equivalence, non-inferiority, or comparative superiority. The absence of between-group differences should therefore not be interpreted as evidence of true clinical equivalence, but rather as an inconclusive finding within the constraints of a small, exploratory cohort. The findings should be interpreted in the context of several key limitations, including limited sample size, absence of formal power calculation, short-term follow-up, and spatially restricted histological sampling. Accordingly, the results are best understood as hypothesis-generating and supportive of further investigation rather than definitive evidence of efficacy or comparability. Future studies with larger populations, rigorous statistical powering, and extended longitudinal assessment are required to more precisely define the clinical efficacy, durability, and potential formulation-specific differences of PLLA-based biostimulation for upper-arm rejuvenation.

## Data Availability

The original contributions presented in the study are included in the article/Supplementary Material, further inquiries can be directed to the corresponding author.

## References

[1] Ao YJ Yi Y Wu GH. Application of PLLA (Poly-L-Lactic acid) for rejuvenation and reproduction of facial cutaneous tissue in aesthetics: a review. Medicine (Baltimore). (2024) 103(11):e37506. 10.1097/MD.0000000000037506 38489708 PMC10939544

[2] Signori R Barbosa AdP Cezar-dos-Santos F Carbone AC Ventura S Nobre BBdS Efficacy and safety of Poly-L-lactic acid in facial aesthetics: a systematic review. Polymers (Basel). (2024) 16(18):2564. 10.3390/polym16182564 39339028 PMC11435306

[3] Arruda S Prieto V Shea C Swearingen A Elmadany Z Sadick NS. A clinical histology study evaluating the biostimulatory activity longevity of injectable Poly-L-lactic acid for facial rejuvenation. J Drugs Dermatol. (2024) 23(9):729–34. 10.36849/JDD.8057 39231078

[4] Nowag B Schäfer D Hengl T Corduff N Goldie K. Biostimulating fillers and induction of inflammatory pathways: a preclinical investigation of macrophage response to calcium hydroxylapatite and Poly-L lactic acid. J Cosmet Dermatol. (2024) 23(1):99–106. 10.1111/jocd.15928 37593832

[5] Triana L Palacios Huatuco RM Campilgio G Liscano E. Trends in surgical and nonsurgical aesthetic procedures: a 14-year analysis of the International Society of Aesthetic Plastic Surgery-ISAPS. Aesthetic Plast Surg. (2024) 48(20):4217–27. 10.1007/s00266-024-04260-2 39103642

[6] Innocenti A Battistella T Gregorio CD Leporati M Luni M Rossati L. Injectable Poly-L-lactic acid (PLLA-SCA™) as a versatile treatment in current aesthetic medicine: expert recommendations based on Italian clinical experience. Cosmetics. (2025) 12(6):264. 10.3390/cosmetics12060264

[7] Christen MO. Collagen stimulators in body applications: a review focused on Poly-L-lactic acid (PLLA). Clin Cosmet Investig Dermatol. (2022) 15:997–1019. 10.2147/CCID.S359813 35761856 PMC9233565

[8] Haddad A Avelar L Fabi SG Sarubi J Somenek M Coimbra DD Injectable Poly-L-lactic acid for body aesthetic treatments: an international consensus on evidence assessment and practical recommendations. Aesthetic Plast Surg. (2025) 49(5):1507–17. 10.1007/s00266-024-04499-9 39592491 PMC11965205

[9] Mazzuco R Evangelista C Gobbato DO de Almeida LM. Clinical and histological comparative outcomes after injections of Poly-L-lactic acid and calcium hydroxyapatite in arms: a split side study. J Cosmet Dermatol. (2022) 21(12):6727–33. 10.1111/jocd.15356 36098704

[10] Vleggaar D Fitzgerald R Lorenc ZP Andrews JT Butterwick K Comstock J Consensus recommendations on the use of injectable Poly-L-lactic acid for facial and nonfacial volumization. J Drugs Dermatol. (2014) 13(4 Suppl):s44–51. 24719078

[11] da Cunha MG Sigrist R. Static and dynamic high-resolution ultrasound analysis of tissue distribution of Poly-L-lactic acid particles during subdermal application in two different presentations. Skin Health Dis. (2023) 3(1):e155. 10.1002/ski2.155 36751337 PMC9892425

[12] Schierle CF Casas LA. Nonsurgical rejuvenation of the aging face with injectable Poly-L-lactic acid for restoration of soft tissue volume. Aesthet Surg J. (2011) 31(1):95–109. 10.1177/1090820X10391213 21239677

[13] Russo PR Bovani B De Angelis F Forte R Vercesi F Salti G. Multicentric retrospective study on safety and efficacy of a novel injectable Poly-L-lactic acid for buttocks recontouring. J Cosmet Dermatol. (2025) 24(1):e16580. 10.1111/jocd.16580 39445541 PMC11743214

[14] Vasconcelos-Berg R Vasconcelos-Berg R Real J Wenz F Avelar LET. Safety of the immediate reconstitution of Poly-L-lactic acid for facial and body treatment-A multicenter retrospective study. J Cosmet Dermatol. (2024) 23(12):3918–23. 10.1111/jocd.16560 39285829 PMC11626369

[15] Wilkerson EC Goldberg DJ. Poly-L-lactic acid for the improvement of photodamage and rhytids of the décolletage. J Cosmet Dermatol. (2018) 17(4):606–10. 10.1111/jocd.12447 29119683

[16] Mazzuco R Dal'Forno T Hexsel D. Poly-L-lactic acid for nonfacial skin laxity. Dermatol Surg. (2020) 46(Suppl 1):S86–8. 10.1097/DSS.0000000000002390 32976175

[17] Li A Fang R Mao X Sun Q. High-Frequency ultrasound for long-term safety assessment of poly-L-lactic acid facial filler. Dermatol Surg. (2022) 48(10):1071–5. 10.1097/DSS.0000000000003548 35834659

[18] Chang S Zhao M Gao W Cao J. Engineered collagen/PLLA composite fillers to induce rapid and long-term collagen regeneration. J Mater Chem B. (2025) 13(3):904–17. 10.1039/D4TB02159B 39659187

[19] Baumann L Bernstein EF Weiss AS Bates D Humphrey S Silberberg M Clinical relevance of elastin in the structure and function of skin. Aesthet Surg J Open Forum. (2021) 3(3):ojab019. 10.1093/asjof/ojab019 34195612 PMC8239663

[20] He T Zhang Z Zhang X Niu H Wang S Wang Q Effects of Poly-L-lactic acid fillers on inflammatory response and collagen synthesis in different animal models. J Cosmet Dermatol. (2025) 24(2):e70000. 10.1111/jocd.70000 39910771 PMC11799711

[21] Ouyang R Liang Y Lu S Zhang Z Wei Q Hu J. Advances in Poly-L-lactic acid injections for facial and neck rejuvenation. Plast Reconstr Surg Glob Open. (2025) 13(8):e7029. 10.1097/GOX.0000000000007029 40765682 PMC12323926

