# Supplementary material for: Poly-L-lactic acid (PLLA) in arm contouring: a prospective and blinded trial of Rennova® Elleva and Sculptra®
Source: Front Surg. 2026 May 8;13:1782368. doi: 10.3389/fsurg.2026.1782368 (PMC13194430; doi:10.3389/fsurg.2026.1782368)
Supplement: Supplementary file 1 [file Supplementaryfile1.pdf]

SUPPLEMENTARY MATERIAL

Supplementary Table 1 . Comparative Analysis of skin elasticity, transepidermal water loss (TEWL), and ultrasound echogenicity (USG Value) for Rennova® Elleva and Sculptra® at 30, 60, and 90 Days. Values are presented as mean ± standard deviation.

**Parameter**		**D30**	**D60**	**D90**
**Skin Elasticity**	Rennova® Elleva	171.6 ± 24.215	186.0 ± 48.132	191.077 ± 28.797
	Sculptra®	173.85 ± 26.615	173.2 ± 52.715	176.615 ± 23.74
**TEWL**	Rennova® Elleva	3.798 ± 1.705	4.682 ± 1.763	3.541 ± 1.657
	Sculptra®	3.72 ± 0.877	4.362 ± 1.879	3.742 ± 1.181
**USG Value**	Rennova® Elleva	1048.95 ± 213.42	927.45 ± 165.366	956.312 ± 187.142
	Sculptra®	1051.4 ± 139.668	977.5 ± 228.221	918.5 ± 202.684
